# Coordinate enhancement of transgene transcription and translation in a lentiviral vector

**DOI:** 10.1186/1742-4690-3-13

**Published:** 2006-02-15

**Authors:** Alper Yilmaz, Soledad Fernandez, Michael D Lairmore, Kathleen Boris-Lawrie

**Affiliations:** 1 Center for Retrovirus Research and Department of Veterinary Biosciences, The Ohio State University, Columbus, OH, 43210, USA; 2 Department of Molecular Virology, Immunology & Medical Genetics, The Ohio State University, Columbus, OH, 43210, USA; 3 Center for Biostatistics, The Ohio State University, Columbus, OH, 43210, USA; 4 Comprehensive Cancer Center, The Ohio State University, Columbus, OH, 43210, USA; 5 Molecular, Cellular & Developmental Biology Graduate Program, The Ohio State University, Columbus, OH, 43210, USA

## Abstract

**Background:**

Coordinate enhancement of transgene transcription and translation would be a potent approach to significantly improve protein output in a broad array of viral vectors and nonviral expression systems. Many vector transgenes are complementary DNA (cDNA). The lack of splicing can significantly reduce the efficiency of their translation. Some retroviruses contain a 5' terminal post-transcriptional control element (PCE) that facilitates translation of unspliced mRNA. Here we evaluated the potential for spleen necrosis virus PCE to stimulate protein production from HIV-1 based lentiviral vector by: 1) improving translation of the internal transgene transcript; and 2) functionally synergizing with a transcriptional enhancer to achieve coordinate increases in RNA synthesis and translation.

**Results:**

Derivatives of HIV-1 SIN self-inactivating lentiviral vector were created that contain PCE and cytomegalovirus immediate early enhancer (CMV IE). Results from transfected cells and four different transduced cell types indicate that: 1) PCE enhanced transgene protein synthesis; 2) transcription from the internal promoter is enhanced by CMV IE; 3) PCE and CMV IE functioned synergistically to significantly increase transgene protein yield; 4) the magnitude of translation enhancement by PCE was similar in transfected and transduced cells; 5) differences were observed in steady state level of PCE vector RNA in transfected and transduced cells; 6) the lower steady state was not attributable to reduced RNA stability, but to lower cytoplasmic accumulation in transduced cells.

**Conclusion:**

PCE is a useful tool to improve post-transcriptional expression of lentiviral vector transgene. Coordinate enhancement of transcription and translation is conferred by the combination of PCE with CMV IE transcriptional enhancer and increased protein yield up to 11 to 17-fold in transfected cells. The incorporation of the vector provirus into chromatin correlated with reduced cytoplasmic accumulation of PCE transgene RNA. We speculate that epigenetic modulation of promoter activity altered cotranscriptional recruitment of RNA processing factors and reduced the availability of fully processed transcript or the efficiency of export from the nucleus. Our results provide an example of the dynamic interplay between the transcription and post-transcription steps of gene expression and document that introduction of heterologous gene expression signals can yield disparate effects in transfected versus transduced cells.

## Background

A challenge inherent to many gene delivery systems is efficient expression of the vector transgene. Enhancement of transcription has been a thoroughly investigated target to improve vector gene expression. For example, introduction of a constitutive viral transcription enhancer or a tissue-specific cellular promoter has been utilized widely to stimulate synthesis of vector transgene RNA [1-4]. In addition to high level synthesis of RNA, efficient post-transcriptional expression is a potent target to improve vector gene expression by maximizing the protein yield per molecule of transgene transcript. Notably, many vector transgenes are complementary DNA (cDNA) copies of the natural intron-containing gene. The elimination of introns is an advantageous approach for reducing of the size of the vector transcript to conform to the packaging capacity of the vector virus. This approach is advantageous in vectors with limited packaging size, as is the case for retroviral vectors [5,6]. However the elimination of intronic sequences can significantly reduce protein yield because the process of splicing promotes the translation of intron-containing genes [7-10]. This activity is attributed to a multiprotein complex that is deposited at exon junctions as a consequence of splicing [11,12]. The elimination of intronic sequences can reduce protein yield in a range of a factor of 2 to 30 [13,14]. Therefore the elimination of introns from a transgene may reduce protein yield per molecule of transgene transcript.

Recently, a unique 5' terminal positive posttranscriptional control element (PCE) was identified in the 5' long terminal repeat (LTR) of two simple retroviruses, spleen necrosis virus (SNV) and Mason-Pfizer monkey virus (MPMV) [15,16]. PCE stimulates translation of non-spliced RNA [16,17]. SNV PCE is a compact 165 nt orientation-dependent RNA element that is composed of two functionally redundant stem-loop structures that present unpaired nucleotides for interaction with the ubiquitous host protein RNA helicase A [[18], T. Hartman and K. Boris-Lawrie, manuscript submitted]. PCE is not strictly position-dependent and sustains activity when repositioned to at least 300 nt downstream of the transcription start site [17]. In addition, PCE facilitates expression of unspliced gag-pol RNA of HIV-1 and the parental retrovirus, SNV [[15], T. Hartman, S. Hull and K. Boris-Lawrie, unpublished].

Results from experiments with cDNA expression plasmids determined that PCE stimulates protein yield from non-spliced mRNA by 7 to 10-fold [17]. Quantitative RNA analysis showed that the increased protein production was not attributable to modulation of steady state RNA level or nuclear export. Rather, the increased protein production was due to increased ribosome association. Additional experimentation determined that PCE does not function as an internal ribosome entry site to stimulate internal initiation on bicistronic reporter RNA [17]. These findings and the determination that PCE requires nuclear interactions for stimulation of translation [19] indicates that PCE is a novel 5' terminal cap-dependent translation enhancer of nonspliced RNA.

In addition to its functional activity, other properties make PCE an excellent candidate for improving translational efficiency of vector transgene mRNA. First, PCE functions in a wide variety of cells in concert with ubiquitously expressed host effector protein. Second, PCE stimulates translation of non-spliced mRNA template, which is a common form of vector transgene mRNA. Third, PCE exhibits flexibility in position relative to the transcription start site, which provides versatility during vector construction. The first goal of this study was to test the hypothesis that SNV PCE increases the translational efficiency of lentiviral vector transgene mRNA. In addition, we reasoned that coordinate enhancement of transgene transcription and translation has significant potential for synergistically improving efficiency of transgene expression in lentiviral vector and in other gene expression systems. The promoter of the lymphotropic SNV is constitutively active in a wide variety of cells types from different species [15,20-22]. The promoter encodes two 46 and 23 base-pair repeats with strong enhancer activity and does not require virus-encoded transcription factor to regulate transcriptional efficiency [21]. We constructed a series of vectors to test whether the combination of PCE and a strong heterologous transcriptional enhancer yields a synergistic increase in protein production. Quantitative analysis of RNA and protein levels were used to characterize the effect of PCE on vector RNA in transfected and transduced cells. The results indicate that PCE and cytomegalovirus immediate early (CMV IE) transcription enhancer function synergistically to significantly improve transgene protein output.

## Results

### CMV IE and PCE function synergistically to increase protein output in transfected cells

**Figure 1:**
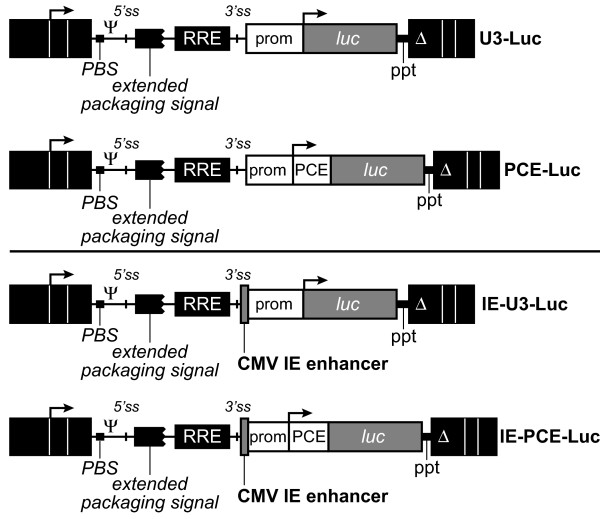
**Genomic structure of self-inactivating lentiviral vectors that lack or contain PCE translation enhancer**. HIV-1 based lentiviral vectors were derived from pHR' [36]. Black rectangles represent HIV-1 long terminal repeats; PBS, primer binding site; Ψ, the core packaging signal; extended packaging signal that corresponds to 350 nt HIV-1 gag open reading frame; 5'ss and 3'ss, splice sites; RRE, Rev responsive element; PPT, polypurine tract; Δ indicates deletion of HIV-1 promoter sequences between -418 to -18; white boxes represent SNV sequences; Prom corresponds to spleen necrosis virus (SNV) U3 promoter; PCE is the 165 nt RU5 region of SNV; CMV IE, cytomegalovirus immediate early enhancer.

A series of HIV-1 based self-inactivating lentiviral vectors were constructed that lack or contain PCE and CMV IE (Figure 1). The vector luciferase (luc) transgene was expressed from an internal transcription unit under the control of the constitutive SNV promoter. The vectors lack or contain SNV PCE and the CMV IE transcriptional enhancer and are designated U3-Luc, PCE-Luc, and IE-U3-Luc and IE-PCE-Luc, respectively.

The vectors were transfected into 293 cells and two days post-transfection, total cellular protein was harvested for Luc assay. Comparison of U3-Luc and PCE-Luc demonstrated that PCE increased Luc activity by 4 to 7-fold (Table 1). Introduction of CMV IE produced a 2.4- to 4.4-fold increase in Luc production (compare U3-Luc with IE-UE-Luc and PCE-Luc with IE-PCE-Luc). Comparison of U3-Luc and IE-PCE-Luc indicated that the combination of PCE and CMV IE produced a cumulative 11 to 17-fold increase in protein production. The results indicate that PCE and CMV IE function synergistically to increase gene expression.

**Table 1:** The combination of PCE and CMV IE increased Luc activity in transfected 293 cells

	Luc activity (Relative Light Units)^a^			
	Replicate Experiment			
	
Vector	1	2	3	4
	
U3-Luc	2,955 ± 171 (1)^b^	3,809 ± 207 (1)	3,605 ± 3 (1)	3,796 ± 700 (1)
PCE-Luc	20,810 ± 559 (7.0)*	19,644 ± 343 (5.1)*	14,756 ± 382 (4.0)*	15,110 ± 842 (3.9)*
IE-U3-Luc	10,490 ± 159 (3.5)	13,174 ± 228 (3.4)	12,945 ± 2,677 (3.6)	16,676 ± 435 (4.4)
IE-PCE-Luc	49,870 ± 28 (16.8)*	48,085 ± 90 (12.6)*	39,485 ± 7,303 (11)*	50,424 ± 1,952 (13)*

^a ^Two-days post-transfection with the indicated vector, which encodes firefly Luciferase (Luc) and Renilla luciferase control plasmid, total cellular protein was harvested and relative Luciferase levels were measured by chemiluminescence assay. Luc level was standardized to cotransfected Renilla Luc and results are presented of four independent experiments performed in duplicate or triplicate. ANOVA with repeated measures determined that increases in response to PCE and IE were significant as indicated by * (p-values of 0.0008 and < 0.0001, respectively).

^b ^(), Fold difference relative to U3-luc vector.

### PCE increases the translational efficiency of lentiviral vector RNA

**Figure 2:**
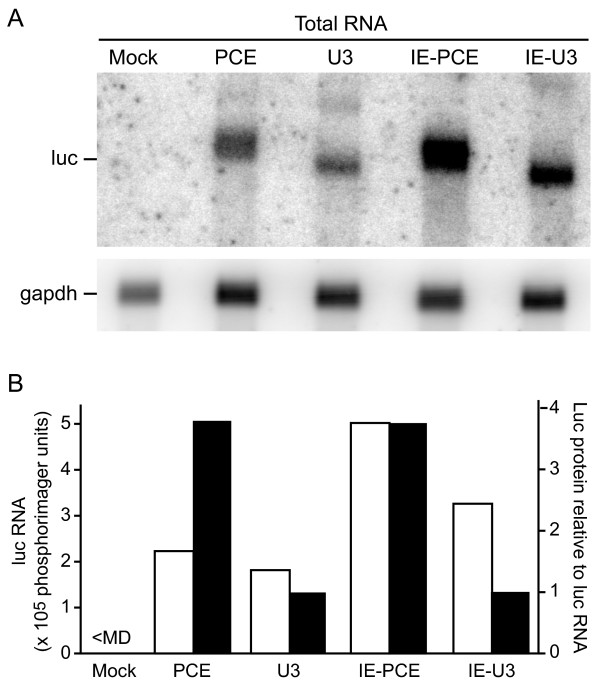
**PCE increases lentiviral vector transgene activity in transfected cells**. (A) Northern blot analysis of total RNA that was isolated 48 hrs after transfection with the indicated vector and hybridized with DNA probe complementary to luc open reading frame or gapdh loading control. The gapdh level was used to standardized minor differences in RNA sample concentration. (B) Graphic representation of data from (A) with white bars indicating the luc RNA levels standardized to gapdh loading control. Black bars indicate translational efficiency relative to the corresponding U3 vector. Translational efficiency is defined as the ratio of Luc activity in these samples to luc mRNA in (A).

Northern blot analysis of total cellular RNA was performed to compare the levels of steady state transgene mRNA. Three replicate Northern blot experiments were performed with radiolabeled probe complementary to the luc open reading frame or glyceraldehyde-3-phosphate dehydrogenase (gapdh) to control for RNA loading. The experiments demonstrated that the vectors express luc transcript of the expected size and that PCE-Luc and U3-Luc displayed similar levels of steady state RNA (Figure 2A). In this representative experiment, luc mRNA levels from PCE-Luc and U3-Luc RNA were 2.2 × 10^5 ^phosphorimager units (PI) and 1.8 × 10^5 ^PI, respectively (Figure 2B). Introduction of CMV IE produced an equivalent 2-fold increase in luc RNA level in either the presence or absence of PCE (IE-PCE-Luc, 5.0 × 10^5 ^PI and IE-U3-Luc, 3.6 × 10^5 ^PI) (Figure 2B). Comparison of the level of Luc protein to luc RNA showed that addition of PCE correlated with a 4-fold increase in Luc protein (Figure 2B). Ribosomal profile analysis determined that ribosome association was greater for the PCE-containing vector than the PCE-lacking vector (data not shown). The results indicate that combination of CMV IE and PCE yielded a synergistic increase in vector transgene expression in the transfected cells.

### CMV IE and PCE function synergistically to increase protein yield in transduced cells

Next we sought to determine whether the coordinate increases in vector transgene expression were sustained in transduced cells. The vector viruses were propagated by co-transfection of 293T cells with each vector, HIV-1 helper plasmid and VSV-G expression plasmid. ELISA was used to measure the capsid Gag levels and equal amounts of Gag were used for transduction by spinoculation of HeLa human fibroblast cells, CEM-A human T cells, D17 canine osteosarcoma cells, and 293 human embryonic kidney cells. Forty-eight hours post-transduction, the transduced cells were harvested and Luc activity in total cellular protein was measured.

The PCE-containing vectors exhibited increased Luc production in all four target cells (Table 2). The magnitude of increase in response to PCE was 2 to 4-fold (Table 2, compare U3-Luc and PCE-Luc). The magnitude of increase in response to CMV IE was an additional 2-fold (Table 2, compare U3-Luc with IE-U3-Luc and PCE-Luc with IE-PCE-Luc). Comparison of U3-Luc and IE-PCE-Luc indicated that the combination of PCE and CMV-IE produced a cumulative 4-fold increase in transgene protein production. These increases were lower in magnitude than the increases observed in the transfected cells (Table 1). Real-time PCR was performed to evaluate provirus copy number and revealed similar levels of vector provirus in transduced 293 cells. In this representative experiment, the copy numbers for PCE-Luc, U3-luc, IE-PCE-Luc and IE-U3-Luc were 4.24 × 10^3^; 6.31 × 10^3^; 3.83 × 10^3^; and 2.07 × 10^3 ^copies/ng, respectively. The results showed that the transduction efficiency was similar between the vectors and was not affected by introduction of PCE or CMV IE.

**Table 2:** The combination of PCE and CMV IE increased Luc activity in transduced cells

	Luc activity (Relative Light Units)^a^			
	Replicate Experiment			
	
Vector	HeLa	CEM-A	D17	293
	
U3-Luc	13,639 ± 2,150 (1)^b^	17,100 ± 524 (1)	18,093 ± 349 (1)	15,099 ± 539 (1)
PCE-Luc	51,998 ± 3108 (3.8)*	52,372 ± 3,354 (3.0)*	50,412 ± 2,997 (2.7)*	27,294 ± 1,173 (1.8)*
IE-U3-Luc	ND^c^	ND	ND	26,197 ± 95 (1.7)
IE-PCE-Luc	ND	ND	ND	59,620 ± 286 (3.9)

^a ^Equivalent vector virus particles were measured by Gag p24 ELISA and 4 × 10^5 ^pg Gag was used to transduce the indicated target cell lines. Total cellular protein was harvested 48 hrs post-transduction and equivalent protein was subject to Luciferase assay. Standard deviations were calculated from results of two or more replicate experiments. ANOVA with repeated measures determined that increases in response to PCE were significant, (p-value of 0.017).

^b ^(), Fold difference relative to U3-luc vector.

### Vector transduction correlates with reduced cytoplasmic accumulation of PCE-Luc RNA

**Figure 3:**
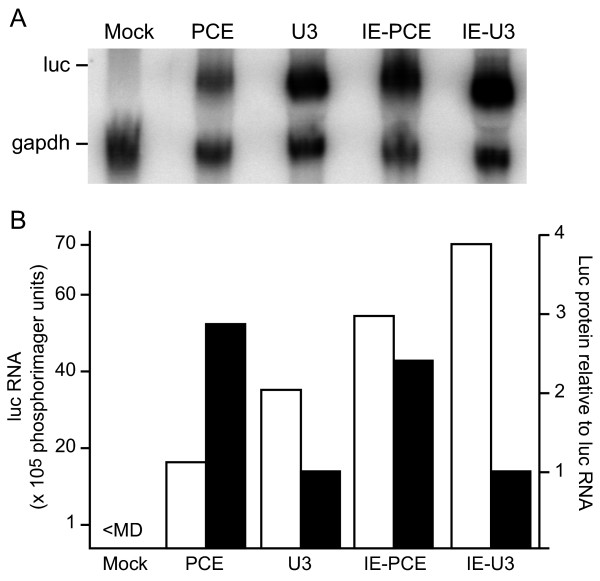
**PCE increases translational efficiency of lentiviral vector transgene RNA in transduced cells**. (A) Northern blot analysis of total cellular RNA that was isolated 48 hours post-transduction and hybridized with a probe complementary to luc or gapdh loading control (B) Graphic representation of the luc RNA in transduced cells. Black bars indicate translational efficiency relative to the corresponding U3 vector. Translational efficiency is defined as the ratio of Luc activity to luc mRNA.

Northern blot assay was used to evaluate steady state luc RNA levels in three replicate experiments. Northern blot analysis of total cellular RNA determined that after transduction, the PCE-containing vectors expressed less steady state luc RNA compared to their PCE-lacking derivative (compare U3 and PCE, IE-U3 and IE-PCE, Figure 3A). Figure 3B summarizes the luc RNA levels standardized to gapdh loading control for this particular experiment. This trend differed from the results in transfected cells, wherein the steady state luc levels were not lower in the presence of PCE (Figure 2). Introduction of CMV IE produced an equivalent 2-fold increase in luc RNA level in either the presence or absence of PCE, which was similar in magnitude to the increase in transfected cells (compare PCE and IE-PCE, U3 and IE-U3, Figure 2). Two of the possible explanations for the lower steady state luc RNA in response to PCE are that PCE lowers the cytoplasmic accumulation or the stability of the luc transcript.

**Figure 4:**
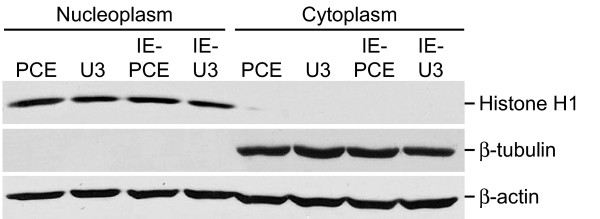
**Western blots demonstrate appropriate subcellular fractionation of transduced cells**. Equivalent amounts of each nuclear or cytoplasmic fraction were subjected to immunoblot with antiserum against the nuclear protein histone H1; the cytoplasmic protein β-tubulin; and loading control β-actin, which is distributed in the nucleus and cytoplasm. The results determined that similar levels of protein were loaded and verified effective subcellular fractionation.

Quantitative analysis of nuclear and cytoplasmic RNA levels was used to investigate possible differences in cytoplasmic accumulation. The transduced cells were fractionated into nucleoplasm and cytoplasm, RNA was harvested and subjected to reverse transcription, and cDNA levels were quantified by real time PCR. Control reactions with actin primers were used to control for minor differences in sample loading. Similar to the Northern analysis of total RNA, the PCE-containing luc RNAs were less abundant in the nucleoplasm and cytoplasm (Table 3, compare U3-Luc with PCE-Luc, IE-U3-Luc with IE-PCE-Luc). Moreover, the accumulation of PCE-Luc RNA in the cytoplasm was lower by a factor of 3. Western analysis was used to verify appropriate fractionation of the nucleoplasm and cytoplasm. Histone H1 was present exclusively in the nuclear fractions and β-tubulin was present exclusively in the cytoplasmic fractions (Figure 4). Immunoblotting with actin verified equivalent sample loading among the samples. The results indicate that PCE-containing luc RNA exhibits lower cytoplasmic accumulation and this difference is proportional to the reduction observed in steady state luc mRNA.

**Table 3:** PCE correlates with reduced cytoplasmic accumulation of luc RNA in transduced cells.

	RNA copy number (× 10^3^)^a^					
			
	Nucleoplasm		Cytoplasm		Cytoplasmic accumulation^b^	Translational efficiency^c^
				
Vector	luc	actin	luc	actin		
			
U3-Luc	56.7 ± 5.4 (1)	59.3	25.3 ± 2.8 (1)	375	0.45	1
PCE-Luc	34.9 ± 3.8 (0.47)	76.6	5.4 ± 0.2 (0.2)	372	0.16	5
IE-U3-Luc	91.3 ± 1.5 (1)	69.1	46.8 ± 12.9 (1)	417	0.51	0.5
IE-PCE-Luc	96.8 ± 2.7 (0.88)	82.5	18.3 ± 2.7 (0.4)	384	0.19	3

^a ^Equivalent amounts (100 ng) of RNA from either the nucleoplasm or cytoplasm were reverse transcribed to generate cDNA and one-tenth of each reaction was quantified by real-time PCR with primers specific to luc or actin. Copy numbers were derived from standard curves generated with pGL3 luciferase plasmid in the range of 10^1 ^to 10^9 ^copies. Reactions were performed in duplicate and the mean and standard deviation are indicated. (), levels of luc normalized to actin relative to PCE-lacking controls, U3-Luc or IE-U3-Luc, respectively.

^b ^Ratio of the copy number of luc RNA in the cytoplasm and nucleoplasm.

^c ^Ratio of Luc activity to copy number of cytoplasmic luc RNA

**Figure 5:**
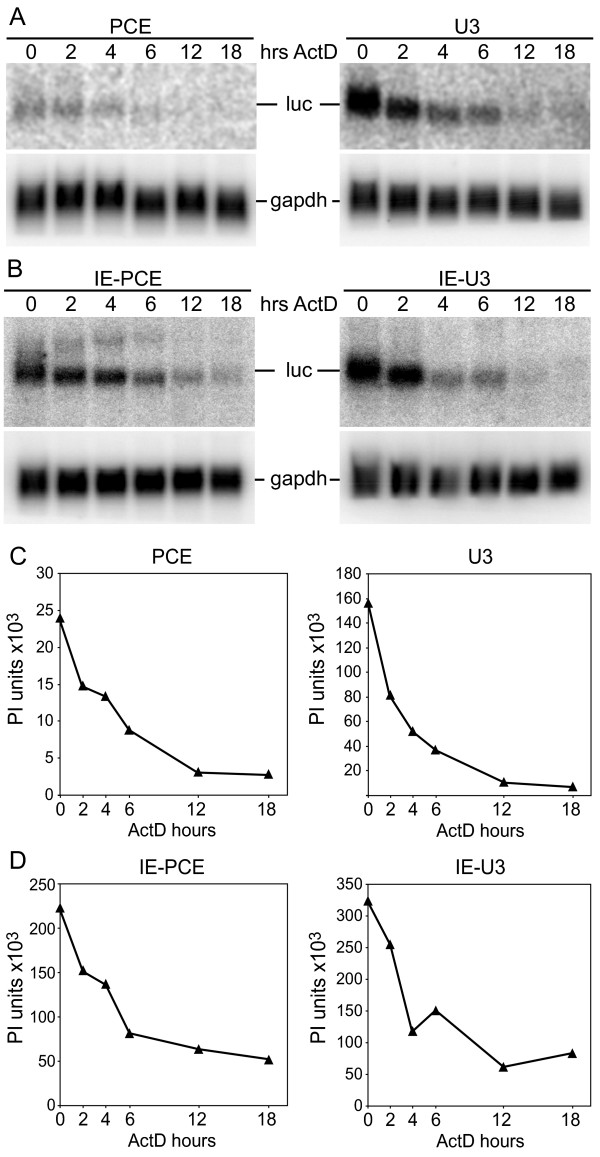
**The half-life of luc RNA is not decreased by PCE**. 293 cells transduced with PCE-Luc, U3-Luc, IE-PCE-Luc or IE-U3-Luc were treated with actinomycin D (ActD) for indicated time intervals and total RNA was isolated and subjected to Northern blot analysis with luc or gapdh complementary DNA probes. (A,B) Northern blot results from a representative of two replicate experiments. The abundance of PCE-containing RNAs is lower than PCE-lacking RNAs, while the abundance of gapdh loading control was similar. (C,D) Decay curves were generated with luc RNA signal standardized to gapdh. The presence of PCE did not reduce the stability of luc RNA.

To investigate the possibility that PCE reduced the stability of the transgene mRNA, the transduced cells were treated with actinomycin D for intervals between 0 and 18 hrs and total cellular RNA was subjected to the Northern analysis. Similar to the Northern analysis shown in Figure 3, the PCE-containing luc RNAs exhibited lower steady state levels compared to the PCE-lacking controls (compare PCE and U3 in Figure 5A, compare IE-PCE and IE-U3 in Figure 5B). In contrast to the differences in luc transcript, the abundance of gapdh loading control was similar among the samples. As shown graphically in Figure 5C and 5D, the decay kinetics of these PCE-containing luc RNAs were no faster than the PCE-lacking control RNAs. The results indicate that PCE did not reduce luc RNA stability. These results taken together with the RT-real time PCR results indicate that the lower steady state level of PCE-Luc RNA is not attributable to reduced RNA stability, but to lower cytoplasmic accumulation. Comparison of the level of Luc activity per molecule of luc RNA present in the cytoplasm indicated that PCE increased Luc protein yield 5 to 6-fold in transduced cells (Table 3). These results indicate that despite the reduction of cytoplasmic accumulation of PCE-luc RNA in transduced cells, PCE translation enhancement activity was sustained. We conclude that the magnitude of translational enhancement is similar in transfected and transduced cells.

## Discussion

Work presented here shows that the PCE can stimulate an increase in lentiviral vector transgene translation. This activity of PCE functioned in synergy with a heterologous transcriptional enhancer and produced a significant 11 to 17-fold increase in gene expression in transfected cells. The presence of PCE is associated with a lower steady state of the transgene mRNA in transduced 293 cells but is not attributable to reduced RNA stability. It is generally accepted that the abundance and localization of an mRNA may be different when expressed from transfected DNA or from an integrated vector in infected cells. An explanation for this observation is that activity of an integrated promoter in a transduced cell is modulated in relation to the local chromatin structure. For example, Williams et al. [23] found that binding of histone deacetylase enzyme HDAC1 to the LTR of an HIV-1 provirus induced alterations in the chromatin structure that disrupted binding of RNA polymerase II and silenced transcription. Additionally, Hofmann *et al*. showed that methylation of the promoter of a lentiviral vector provirus led to transcriptional inactivation [24]. A possible explanation for the lower steady state level of PCE-Luc RNA in our transduced cells is reduced transcription attributable to promoter methylation. A further consideration is that our Northern blot and RT-real time PCR results indicate that the lower steady state PCE-Luc RNA was attributable to post-transcriptional modulation.

It is now clear that steps in transcriptional and post-transcriptional control of gene expression are functionally and physically linked [25]. For example, cotranscriptional interaction with nuclear RNA processing factors is mediated by the carboxy-terminal domain (CTD) of the largest subunit of RNA polymerase II [26-29]. The CTD choreographs deposition of multiprotein complexes on nascent pre-RNAs that implement efficient export from the nucleus and translation in the cytoplasm [25,27,28]. The multisubunit TREX complex, which is conserved from yeast to man, links the apparently distinct processes of transcription and mRNA export [30]. Biochemical analysis of TREX has identified interaction with both intronless and intron-containing genes and determined a relationship between its cotranscriptional recruitment and pre-mRNA retention [31]. Furthermore, the process of transcription is linked with mRNA 3' end formation. RNA polymerase II elongation complexes undergo multiple transitions at the 3' end of genes [27,32,33]. An exchange of elongation and polyadenylation/termination factors at the 3' end of genes choreographs efficient transcription termination and polyadenylation. Alteration in the transcriptional activity of the promoter may invoke unexpected effects on 3' end formation and reduce the steady state mRNA. Based on our results of Northern and RT-real time PCR RNA analysis we speculate that incorporation of the vector provirus into chromatin altered the cotranscriptional deposition of nuclear factors on the nascent PCE-Luc RNA that mediate efficient 3' end formation or nuclear export. Our analysis also determined that the stimulatory effect of PCE on translation activity was sustained despite less efficient upstream steps in gene expression. This observation suggests that factors necessary for PCE translation stimulation remained available despite less cytoplasmic accumulation.

Our study using the luc transgene suggests that changes in early steps in ribonucleoprotein particle formation profoundly influenced the availability of mRNA available for translation enhancement by PCE. We project that the activity of PCE and CMV IE to co-ordinately stimulate protein output will be sustained in other transgenes. However, the unique features of any particular transgene s likely to influence the efficiency of 3' end formation or other post-transcriptional process [33,34]. Furthermore, vector integration is expected to induce epigenetic modulation of gene transcription that may profoundly affect the absolute level of RNA available for protein synthesis. The results of our study are consistent with the recent realization of tight linkage between the transcription and post-transcriptional steps in gene expression and emphasize the important role epigenetic modulation plays in vector gene expression.

## Conclusion

Coordinate enhancement of transgene transcriptional and post-transcriptional expression represents a potent approach to increase transgene protein production in a broad array of gene expression systems, including lentiviral vectors, other viral vectors and non-viral gene expression plasmids. Our results show that combined introduction of the SNV PCE 5' terminal translational enhancer and CMV IE transcriptional enhancer to HIV-1 based lentiviral vectors significantly improved protein yield per molecule of intronless transgene RNA in transfected cells and in four transduced cell lines. Increasing the protein yield per RNA molecule is expected to be a useful approach in a diversity of gene expression systems. This approach could compensate for limited promoter activity observed in some in vivo studies wherein strong promoter activity was not achievable using a tissue-specific promoter [35].

## Methods

### Plasmid construction

The Luc vectors were derived from self inactivating pHR'CMV-GFP [36]. First, we constructed HIVSIN-Luc by insertion of a linker (5'TCGATGGATCCACTAGTC 3' and 5'TCGAGACTAGTGGATCCA 3') into the XhoI site in pHR'CMV-GFP thereby introducing an SpeI site. GFP was replaced with the luc open reading frame in the BamHI-XbaI fragment from pCAM-Luc by ligation into BamHI and SpeI. pCAM-Luc was constructed by insertion of a PCR product containing luc from pGL3 (Promega, Madison, WI) with BamHI and XbaI termini into pPCR-Script CAM SK+ (Stratagene, La Jolla, CA). PCE-Luc was derived from HIVSIN-SNVLTR-GFP and U3-Luc was derived from HIVSIN-SNVU3-GFP. To construct HIVSIN-SNVLTR-GFP and HIVSIN-SNVU3-GFP, the NdeI-BamHI fragment from pHR'CMV-GFP was replaced with the NdeI-BamHI fragment that contains the SNV U3RU5 or U3 sequences of pYW100 and pYW205 [15], respectively. In order to replace the GFP fragment in HIVSIN-SNVLTR-GFP and HIVSIN-SNVU3-GFP with the luc open reading frame, DNA oligonucleotides (KB973–KB974) were annealed and ligated a the XhoI site in each vector and then NdeI-SpeI fragment that contains SNV U3RU5-Luc and SNV RU5-Luc from pSNVRU5*luc *and pSNV*luc *[17] was inserted to create PCE-Luc and U3-Luc, respectively. The CMV-IE enhancer region from pRL-CMV (Promega, Madison, WI) was amplified by PCR with primers (5'TTTTTATCGATAAGCTCAATATTGGCCATATTATTCATTGG3' and 5'TTTTCATATGCAGTTGTTACGACATTTTGGAAAG3') and ligated with NdeI-ClaI-digested PCE-Luc and U3-Luc in order to create IE-PCE-Luc and IE-U3-Luc, respectively.

### Transient transfection and Luciferase assay

Transient transfections were performed on 2 × 10^5 ^293 cells in duplicate 60 mm plates. Five μg vector DNA and 0.5 μg pRL-CMV (Promega, Madison, WI) were cotransfected by CaPO_4 _method [15]. The cells were harvested in PBS at 48 h post transfection, centrifuged at 1500 × *g *for 3 min and resuspended in 150 μl of ice-cold NP-40 lysis buffer (20 mM TrisHCl [pH 7.4], 150 mM NaCl, 2 mM EDTA, and 1% NP-40). Dual-Luciferase reporter assay (Promega, Madison, WI) was performed with 10 μl lysate, 100 μl Luciferase assay reagent II (Promega, Madison, WI) and 100 μl Stop&Glow™ (Promega, Madison, WI) according to manufacturer's protocol and quantified in a Lumicount luminometer (Packard Instrument Company Inc., Downers Grove, IL). The level of Ren activity was used to standardize transfection efficiency. Luc activity is presented relative to Ren activity. Luc activity in transduced cells was also determined 48 hours after transduction by the same procedure. Cells were lysed in 100 μl ice-cold NP-40 lysis buffer and Luc assay was performed with 10 μl lysate and 100 μl Luciferase assay reagent (Promega, Madison, WI).

### Preparation of vector virus stocks and transduction

Lentivirus vector stocks were produced by transient triple transfection of 293T cells with 10 μg of HIV-1 gag-pol packaging plasmid pCMVΔR8.2 [37], 2 μg of the pMD.G VSV glycoprotein expression plasmid [37], and 10 μg of the vector plasmid by the CaPO_4 _method [15]. After overnight transfection of 5 × 10^6 ^293T cells in a 10-cm plate, the cells were cultured in fresh DMEM (Invitrogen, CA), 10% FBS and 10 mM sodium butyrate for 8 hours. The supernatants were collected at 12 hour intervals over a 60 hour time period and passed through a 0.2-μm filter (Corning, NY) and concentrated by ultracentrifugation at 80,000 × g at 24°C for 2.5 h in a Beckman SW28 rotor. HIV-1 Gag concentration was determined by Gag p24 ELISA (Coulter, Hialeah, FL). 293, HeLa, CEMx174 and D17 cells were transduced with 4 × 10^5 ^pg Gag in 6-well plates by spinoculation at 1500 × g for one hour at 32°C [38]. Spinoculation of 293 cells was performed in the presence of 8 ug/ul polybrene.

### RNA preparation

Total RNA was isolated from approximately 5 × 10^5 ^cells in 0.5 ml Trizol reagent (Invitrogen, CA) according to manufacturer's protocol. Cells were treated with 5 ug/ml actinomycin D for 2, 4, 6, 12 and 18 hours. To harvest nuclear and cytoplasmic RNA, a subconfluent 100 mm plate of cells was incubated with hypotonic lysis buffer (10 mM HEPES [pH 7.9], 1.5 mM MgCl2, 10 mM KCl, 0.5%NP40, and 0.5 mM dithiothreitol) for 10 min on ice andsubjected to two rounds of centrifugation at 3000 × g for 2 mins at 4°C. One-tenth of the nuclear pellet and cytoplasmic supernatant were reserved for Western blotting. The pellet was treated with Trizol (Invitrogen, CA) and the supernatant was treated with Trizol-LS (Molecular Research Center, Cincinnati, OH) and RNA was extracted by the manufacturer's protocol.

### RNA analysis

For Northern blot analysis, 5 μg total RNA was separated on 1.2% agarose gels containing 5% formaldehyde, transferred to Duralon-UV membrane (Stratagene, La Jolla, CA), and incubated with either luc or gapdh DNA probes. The probes were prepared by a random-primer DNA-labeling system (Invitrogen, CA) with gel purified luc or gapdh restriction products and [α-^32^P]dCTP. The hybridization products were scanned with PhosphorImager (Molecular Dynamics, Sunnyvale, CA) and quantified by ImageQuant software (Molecular Dynamics, Sunnyvale, CA). Sucrose gradients were prepared from 1 × 10^7 ^293 cells in a T150 flask 48 hours post-transfection as described previously [17] and isolated RNA was subjected to Northern blot.

For reverse transcription, random hexamer and Sensiscript reverse transcriptase (Qiagen, Germany) were used to generate cDNA from 100 ng of cytoplasmic or nuclear RNA. Ten percent of the cDNA preparation was used for real-time PCR with primers complementary to luciferase or actin and Quantitect SYBR Green PCR (Qiagen, Germany) in a Lightcycler (Roche, Germany). Copy numbers were derived from standard curves generated with pGL3 luciferase plasmid in the range of 10^1 ^to 10^9 ^copies. Reactions were performed in duplicate and the mean and standard deviation are presented.

### Western blotting

Bradford assay was used to measure 50 μg of protein from nuclear and cytoplasmic fractions. Proteins were separated by SDS-PAGE and transferred to nitrocellulose membrane. Immunoblotting was performed with mouse monoclonal antibodies against histone H1, β-tubulin and β-actin (Abcam, Cambridge, MA). Visualization was performed with Luminol reagent (Santa Cruz Biotechnology, Santa Cruz, CA).

## Competing interests

The author(s) declare that they have no competing interests.

## Authors' contributions

AY conceived of the study, carried out the vector construction, experimental evaluation, and participated in the data analysis and preparation of the manuscript. SF participated in the design of the study and carried out the statistical analysis. MDL participated in the preparation of the manuscript. KBL coordinated the design and implementation of the study, the data analysis, and the preparation of the manuscript. All authors read and approved the final manuscript.
